# Development, Responsibility, and the Creation of Urban Hazard Risk

**DOI:** 10.1177/15356841211046265

**Published:** 2021-09-27

**Authors:** Timothy J. Haney

**Affiliations:** 1 Mount Royal University, Calgary, AB, Canada

**Keywords:** disaster, risk, development, hazards, place

## Abstract

Scholarly attention has recently shifted to the creation and redevelopment of urban hazardscapes. This body of work demonstrates how housing is deployed in close proximity to hazards, and how the attendant risks have been communicated—or not—to potential residents. Utilizing the case of Calgary, Alberta, this article uses interview data collected from flood-impacted residents, and looks at their perceptions of development and risk creation. The analyses focus on how people attribute responsibility for development in flood-prone areas, and their views on future development in these areas. Results reveal that many residents argued for more government regulations preventing new development in floodplains. Moreover, they viewed developers as narrow-interested capitalists who fail to protect public safety and work to conceal risk from the public. Others wished to see large structural mitigation projects—dams, levees, or floodwalls—or insisted that homebuyers be informed of flood risk prior to purchase. The article concludes by addressing the implications for scholarly work in urban sociology, environmental sociology, and the sociology of disaster—all of which grapple with tensions between place-making and risk creation.

## Introduction

The new neighborhood of Riverstone, in Calgary, Alberta boasts on its website that the community is “surrounded by 360 acres of environmental reserve” with “lots backing onto greenspace and Fish Creek Park” (Calbridge Homes 2019). Brookfield Residential, the real estate firm charged with selling the Riverstone properties, tells buyers it is a “community on the edge of the Bow River that features a beautiful natural escape, thoroughly designed streetscapes and stunning views, all within city limits.” Thanks to twenty-first-century flood mitigation, “your family can safely enjoy all the advantages of living beside the beautiful Bow River year-round, without concerns” (Brookfield Residential 2019), though this statement about concerns was subsequently removed. Bucolic as it sounds, Calgary’s regulatory flood maps reveal that Riverstone was built almost entirely within the city’s Flood Fringe, designated by engineers and hydrologists as a high-risk flood area.

The case of Riverstone illustrates a pattern across North American cities. Rivers and streams provide opportunities for outdoor recreation and picturesque living. But they also carry the risk of flooding, as Calgary saw in 2013 when the Bow and Elbow rivers overtopped their banks, forcing the evacuation of more than 75,000 people, and causing more than $6 billion in damages. Yet following this destruction, Calgary has continued developing in flood-prone areas, owing largely to developers’ political and economic clout. Similar developments have been built across Canada, the United States, and the United Kingdom (Bruemmer 2019; Mazur 2019; Melnychuk 2019; Rowlatt 2019). Research often focuses on the tactics of a city’s “growth machine,” a body of work that too often overlooks resident voices, risk framings, and concerns. As such, nearly absent from discussions of the pros and cons of developing in floodplains are the views and concerns of residents themselves—who engage in a particular politics of homeownership and placemaking, while at the same time working to safeguard their place attachment and property values (McCabe 2016). More broadly, although there are some iconic case-studies of environmental injustices that affect marginalized communities, residents of urban hazardscapes are less commonly asked about how they view the ongoing production of risk.

To add this missing piece, this article applies a theoretical framework drawn from the political economy of place-making, urban development, and risk, to answer several questions: How do residents living in flood-prone areas attribute responsibility for the creation of risk and protection from flooding? How do residents view developers and the municipal government? Does experiencing a disaster prompt flood-prone residents to ask for more regulation of the development and home-building industry? Do residents favor requirements that real estate agents and/or developers disclose a home’s location in a flood-prone area to potential buyers? Finally, to what extent do they prefer structural mitigation efforts?

Using interviews from 40 residents living near Calgary’s two rivers, this article begins a conversation about flood risk and responsibility. As nearly unabated development of flood-prone areas continues, this analysis reveals how residents understand these new forms of risk. It explores how residents in a city with a powerful local growth elite and continuing development near hazards view land use regulations in the wake of a catastrophic flood that threatened their livelihoods and property values.

As the analysis reveals, participants voiced many views about who should shoulder responsibility for halting the creation of new flood risks, and what approach is best. Many placed the onus on government, either to limit development near rivers or to mandate that homebuyers be informed of risk. They expressed openness to regulations, particularly when flood risk threatened home values. Others preferred an individualistic “buyer beware” approach or asked for enhanced structural mitigation efforts—dams, levees, or floodwalls. These different approaches, as we shall see, signal a lack of consensus about responsibility and about best-practices for flood risk mitigation.

## Literature Review

Early twentieth-century urban sociologists examined how parcels of land changed as they were bought, sold, developed, or abandoned. The Chicago School of urban sociology also demonstrated how particular ethnic and socioeconomic groups moved into and out of a space over time (see Hartmann 1993; Schwirian 1983 for reviews). It advanced a human ecological model of the city outlining how the provision of housing and urban amenities map onto changing neighborhood and local demographics (Park 1952). Most importantly, it demonstrated that social relations do not occur aspatially nor on the head of a pin, but are emplaced in both space and time (Abbott 1997).

Later in the century, urban political economists added a layer of complexity, demonstrating land-use decisions as products of competition for resources, profit motive, and negotiation of different parties (Harvey 1985; Katznelson 1982). This approach outlined how urban elites transform space to serve their interests. Only recently have we studied how these processes of migration and development also create an urban landscape of risk. Due to cities’ capital-intensive infrastructure, population concentration, and ubiquitous hazards (Fu 2016) residents must be protected from risks amid capitalist expansion. The following section analyzes these complex interactions by exploring the role of development as a driver of urban risk creation.

### The Political Economy of Urban Placemaking

Recent work highlights how urbanization operates as a dialectic process of systemic risk production and obfuscation. Development in risky areas is carried out by a local network of builders, developers, investors, real estate interests, and other incentivized parties. Typically, the state initiates the process of landscape transformation but then turns the process over to members of this growth coalition (Rudel 2009). With the blessing of the state, parties actively push growth into hazardous urban spaces, including along waterways (Nicholls and Crompton 2017), in low-lying coastal areas (Brody, Kim, and Gunn 2013), on fault lines (Ramseyer 2012), and near landfills (Gaffney 2018). The potential costs associated with developing in risky places are now included in developers’ calculations (Fu 2016). The network of pro-growth interests “captures” local political officials and works to undercut existing environmental protections (Clement and Elliott 2012). Municipal governments are particularly vulnerable to being “captured” because the responsibility for regional development has largely devolved from the federal to the municipal level, making these dynamics more susceptible to advocacy from pro-growth actors (Rudel 2009).

Despite the attractive logic of limiting development in flood-prone areas, many scholars note the barriers for doing so. One key problem is the “extent to which local government is pressured by economic development needs, land scarcity, or both, to allow development in, or very near, the 100-year floodplain” (Birkland et al. 2003). In Calgary, recordings from 2013 reveal a discussion among developers about how to wield power over city council, with one builder bragging about fundraising for candidates who support development (Vaessen and Elliott 2013; Walton 2013). Exacerbating this pressure, homeowners often understand property rights as absolute, resisting municipal decisions to limit or regulate development. In turn, local governments “may shy away from adopting stringent land use codes for fear of future legal objections and a potential backlash from voters” (Brody, Highfield, and Kang 2011:84; see also Tarlock and Albrecht 2018).

When implemented, land use regulations limiting or prohibiting development have been successful in slowing residential development near hazards (Jackson 2016), while at the same time, risk-sensitive planning has proven cost-effective for governments (Sudmeier-Rieux et al. 2015). However, conservative communities often oppose all regulations, fearing overreach by “big government” even when those regulations may benefit them, or when they already benefit substantially from existing government programs (Hochschild 2016). This objection to government regulation often occurs because residents see risk as the inevitable (Lupton and Tulloch 2002) tradeoff for economic growth.

The classic essay “The City as a Growth Machine” (Molotch 1976; revised as Logan and Molotch 1987) explains how a city’s “growth elite” (including builders and developers as central players) clamors for continued growth, despite the environmental costs. Those in the position to benefit from growth “encourage growth . . . for its own sake” (Logan and Molotch 1987) through “incessant lobbying, manipulating, and cajoling” (p. 52). In a recent iteration of this logic, with an eye on hazard creation, Tierney (2014) argues:

Pro-growth actors . . . prefer to operate in environments in which they are not constrained by land use plans, zoning and code requirements, and environmental regulations. They generally oppose regulations and requirements that would add to the cost of building and infrastructure construction and maintenance . . . and they seek to comply only minimally with existing mandates. Second, growth machine coalitions deemphasize the risks associated with places and spaces, focusing instead on the amenities provided by these locations. (127–28)

Developers have few incentives to focus on risks, as those who profit from development activities in risky locations in the short term do not bear the ultimate costs; individual property owners, communities, insurance providers, and taxpayers bear those costs (p. 128). This is true in Calgary, where home builders shoulder financial liability for one year after completion, part of a mandated warranty on new homes (Province of Alberta 2013). After one year, financial risk is transferred to the homeowner. In most North American cities, developers “will be protected from liability by legal walls that are much closer to being watertight than are the levees” (Freudenburg et al. 2008:1023). This is of particular concern given that until 2015, Canadians could not purchase flood insurance; only now are a small number of people beginning to carry coverage (Henstra et al. 2019; Thistlethwaite 2017; Thistlethwaite et al. 2017). In this context, developers quickly transfer risk off their ledgers and onto homebuyers.

### Resident Views of Risky Development

Given the ubiquity of residential development in North American cities, and the increase of new developments in flood-prone areas, we might expect a robust literature on resident attitudes toward floodplain development. Unfortunately, this is not the case. Most literature on resident views explores development of tourism infrastructure (Long and Kayat 2011; McNicol 2004), transit (Noland et al. 2017), and sports facilities (Bob and Swart 2009; Scherer 2016), but not housing. New and emergent work in urban sociology looks at how relic industrial sites breed hazards that become obfuscated as land uses change over time (Elliott and Frickel 2015; Frickel and Elliott 2018). Little work has examined how residents perceive the ongoing creation of new risks, particularly the construction of housing near natural hazards. Although scholars pay much attention to expert understandings of risk, they pay significantly less to layperson understandings of risk, often cast as uninformed (Rothstein 2003) or overly emotional (Lupton 2013). There are, however, a few notable exceptions.

To begin, extant literature provides some perspective on how residents view developers. This small body of work reveals that residents support development, but hold negative appraisals of developers (Monkkonen and Manville 2019), as they resent the tremendous profits that developers stand to make. Work by Morris-Oswald and Sinclair (2005) echo this sentiment, demonstrating how residents value cooperation but feel that riverine management has largely excluded them from the conversation, as it favors technocratic, rather than democratic, approaches (Glenna 2010). Furthermore, while some publics distrust government and risk-regulation experts uncritically, others reject expert knowledge more critically (Poortinga and Pidgeon 2003).

To challenge ongoing risky development practices, residents must have scientifically accurate understandings of flood risk. One obstacle to this understanding, however, is that flood maps and hydrological assessments are often out of date (Stevens and Hanschka 2014) and that the metric used (i.e., the 1-in-100 year flood zone) inadequately captures real levels of risk (Highfield, Norman, and Brody 2013). Nor do maps account for the ever-changing landscape of risk, particularly in coastal regions heavily affected by climate change (Whitney and Ban 2019). In the case of riverine flooding, peak flows vary and are irregularly calculated (Jakob and Church 2011), leaving scientists with only a vague approximation of risk. Even when scientists hold an accurate understanding of risk, the way they communicate (couched in scientific language) often fails to answer residents’ most fundamental question: “am I safe?” (Frickel and Vincent 2007). This results in a verifiable gap in awareness of flood risks between experts and the public (Chowdhury and Haque 2011).

Attention has shifted recently to “room for the river” approaches that either restrict development in floodplains (Bogdan, Beckie, and Caine 2021; de Groot and de Groot 2009) or offer buy-outs for residents to move from risky areas (Koslov 2016; Zavar 2015). Do residents prefer these approaches? Although research into resident views is scarce and the literature is fairly split, it teaches us a few lessons. Dutch residents, despite holding eco-centric ideals, resist the notion that areas immediately adjacent to rivers should be left undeveloped to allow “room for the river” (de Groot and de Groot 2009). In contrast, residents generally have positive views of buyout programs in flood-prone areas, and see the resultant green space as a desirable environmental amenity (Zavar 2015). Related research demonstrates that these “gain frames” (i.e., amenities) are more effective for encouraging risk mitigative behavior than “loss frames” (i.e., fear of flooding) (Spence and Pidgeon 2010). The literature examining public views of development and land-use decisions is also rooted in discussion of space and place-attachment. Both security gained through structural mitigation, and amenities such as green spaces, are key elements for placemaking and strong place-based social capital (Coaffee 2013; Mattijssen et al. 2017; Wu and Hou 2020).

In Canada, the least resistance to floodplain regulation comes from residents most aware of their own residential flood risk (Kreutzwiser, Woodley, and Shrubsole 1994). Residents feel more committed to places where they feel protected from hazards and have access to green space and amenities, both of which can be augmented through “room for the river” approaches. Yet, Larson and Santelmann (2007) find that resident preferences for environmental preservation are directly related to proximity to water. This attitude probably relates to perceived risk, as previous research has found that proximity to hydrological hazards is related to flood risk perception (Gray-Scholz, Haney, and MacQuarrie 2019).

Efforts to resist development perhaps best demonstrate this resident opposition (Gotham 2011; Lees and Ferreri 2016; Scherer 2016). But how efficacious is public participation for resisting the imposition of risks? Adua and Lobao (2021) show that the involvement of citizen groups and nongovernmental organizations in local governance is statistically unrelated to the adoption of controls on growth (wetland protections, urban growth boundaries, etc.). Even the influence of business is surprisingly circumscribed, but it nevertheless dwarfs the effectiveness of public participation on land use decision-making, often because developers purchase the consent of the community through donations and sponsorships (Garboden and Jang-Trettien 2020).

Other research indicates that municipal governments, though closely aligned with development interests, are responsive to grassroots citizen engagement. Municipalities are more likely to grant building permits in hazard-prone places, and do so more quickly, where there is local political participation (Go 2014). Combined with public unpopularity, land-use regulations also predict decreased rates of growth in and around a municipality (Jackson 2016). Owing to these dynamics, planners in Calgary struggle to curb developers’ ambitions who “set the agenda in Calgary,” as in many cities (Grant 2009:20). In this “hegemonic growth regime,” “the most active group (in most cases the Chamber of Commerce and/or real estate developers) favors growth, and all other active groups—including neighborhood associations—are pro-growth or neutral,” meaning there are few voices opposing unabated growth (Logan and Crowder 2002).

All of this begs the question: If both the “growth elite” and residents oppose restrictions on development, what approaches to risk mitigation *do* residents prefer? In conservative neoliberal cities like Calgary, we might expect residents to favor “personal responsibility” over government regulation. As Henstra et al. (2019) find, residents are willing to accept *some* responsibility for flood risk mitigation, but the small share of responsibility they take is insufficient to influence their decisions, such as by adopting property-level flood-mitigation practices (sump-pumps, back-flow preventers, etc.) that have been found to effectively attenuate aggregate flood losses (Kreibich and Thieken 2009).

The widely preferred approach today is structural mitigation—the building of berms, levees, dikes, pumps, and floodwalls—to protect people and property. This approach reflects “the idea that people can use technology to control nature to make themselves safe” (Mileti 1999:2). Although structural mitigation can decrease flood damage in the aggregate, other methods have proven more effective (Brody, Zahran, Maghelal, Grover, and Highfield 2007). The growth lobby favors these increasingly sophisticated structural mitigation techniques (Alexander 2000:25; Lara et al. 2017; Vari, Linnerooth-Bayer, and Ferencz 2003), and the public frequently prefers these mega-infrastructure projects because of the psychological assurance and the political payoff they provide (Bogdan et al. 2020).

Despite bureaucrats and developers preferring this approach, residents sometimes prefer property-level, non-structural risk mitigation methods (Rasid and Haider 2002). Furthermore, residents tend to like engineered infrastructure for risk mitigation (Gray, O’Neill, and Qiu 2017), suggesting again that limitations on residential development may not be preferred. There are also temporal complexities with structural mitigation; although the costs are borne in the present, the benefits accrue only over the long-term, therefore seeming uncertain (Henstra 2012) and conferring a “present bias” against planning for possible future events (White and Haughton 2017).

There is also evidence that residents want a “buyer beware” approach to risk mitigation, but coupled with widely disseminated information about risks. Shrubsole and Scherer (1996) find widespread agreement by Canadian real estate agents and floodplain residents that potential buyers should be informed of a home’s flood risk. This agreement results largely from the shared knowledge that floodplain location does not negatively impact property values in a context where residents expect government to protect their property values (Becher 2015).

Perhaps the most compelling time to look at land-use decisions and development comes soon after disaster. While we might expect flood disasters to curtail future development plans, Elliott and colleagues demonstrate how disasters encourage more intense land-use development and demographic growth (Elliott and Clement 2017; Elliott and Pais 2010; Pais and Elliott 2008; Schultz and Elliott 2013). As this development unfolds, discussions of the priorities of the various parties involved in hazard creation and mitigation (developers, builders, municipal governments, residents) become all the more salient, and a dynamic movement exists between conflict and cooperation (Becher 2010). The dynamic tensions, the power of developers, and a public at least partially unsure about best practices for decreasing flood risk converge to make hazard-affected residents an unlikely source of resistance.

There are several looming unanswered questions: How do residents in a city with a powerful local growth elite and continuing development near hazards view land use regulations in the wake of a catastrophic flood that threatened their livelihoods and property values? Is that experience enough to prompt them to call for greater regulation? What strategies of risk mitigation do they prefer, and how do they understand responsibility for risk creation and abatement? These questions are particularly germane in Calgary a wealthy, fossil-fuel dependent city where the public normally rejects government restrictions on private property rights.

### Risk and Disaster in Alberta

Calgary occupies a strategic riverine location at the confluence of the Bow and Elbow Rivers (Pomeroy, Stewart, and Whitfield 2016). The larger of the two, the Bow, originates from the Blow Glacier in the Canadian Rockies, and flows into the city from the west, eventually flowing through many of the city’s oldest neighborhoods, meeting the Elbow near Fort Calgary. After this meetup, the Bow flows southward, through many newer communities like Riverstone, Cranston, and Chaparral.

In 2013, the Bow and Elbow overtopped their banks, causing the evacuation of 75,000 people, and resulting in $6 billion in damages (Gandia 2013). Both groundwater intrusion and sewer backup inundated thousands of homes (Abboud, Ryan, and Osborn 2018). Events like the 2013 flood are poised to become more common, as Alberta is projected to warm at more than twice the global rate—as much as 4 degrees Celsius by 2050 (Sandford and Freek 2014). Already accounting for 60 percent of Canada’s total disaster damage since 2010 (Edwardson 2018), future claims to the federal government’s Disaster Financial Assistance Arrangements from Alberta are projected to increase substantially (Parliamentary Budget Officer 2016).

Since the 2013 flood, Calgary, with financing from both the provincial and federal governments, completed upgrades to storm sewers, installed new flood barriers, upgraded existing dams and reservoir capacity, subsidized property-level measures like backflow preventers, and launched community education initiatives, spending more than $318 million by 2023 (City of Calgary 2020). Development of new residential communities, nonetheless, continues along the river on the southern side of the city (see Riverstone in Introduction). But recent efforts to halt urban sprawl also have pushed redevelopment inward to the city’s older river-adjacent communities, allowing builders to raze existing homes and build two (or more) in their place. Known locally as “infills,” those homes mean that the population is also growing in existing neighborhoods near the rivers, not solely on the outskirts. Unlike many North American cities, where the wealthiest denizens protect themselves from flooding by purchasing property far from or higher than bodies of water, in Calgary the wealthiest people buy property *near* hazards because of amenities like scenery and recreation (Bolin and Kurtz 2018:193), which have been found to increase property values (Bourassa, Hoesli, and Sun 2004). Residential growth is projected to continue and expand as Calgary envisions growing its current population of 1.3 million to 1.7 million by 2033, and to more than 2.4 million by 2076 (Calgary Metropolitan Region Board 2018).

Alberta is known as the most conservative Canadian province. Its history—celebrated annually at the Calgary Stampede, the largest rodeo on earth—touts rugged individualism embodied by self-made oil-men, cowboys, and ranchers. This history makes Calgary and Alberta particularly prone to right-wing populism (Davidson 2019). Albertans maintain individualistic values, seeing a very limited role for government. They “generally favor governments and political leaders capable of protecting an enviable quality of life by keeping taxes low” and eschew “activist government” (Sayers and Stewart 2019). Albertans therefore prefer deregulation for the provision of services such as electricity (Woo et al. 2012) and water (Nicol and Klein 2006), while ardently defending the sanctity of private property rights (Evans and Garvin 2009). Given this context, how do flood-prone residents, having recently been through a flood event, view responsibility for risk creation and mitigation; what strategies of risk mitigation do they prefer? To get at the dynamics discussed above, the present study focuses on a neoliberal city, where flood risks have been systematically produced over time, and where this risk has recently resulted in catastrophic urban flood events.

## Data and Methods

The article uses qualitative data from 40 in-depth interviews with flood-affected residents, which took place in the Fall of 2015. Recruitment of these participants was done through community associations in Calgary’s 26 flood-affected neighborhoods (City of Calgary 2018). An e-mail was first sent to officials within each community association, asking for help recruiting participants. Community association leaders then forwarded the e-mail to members, discussed the interviews at meetings, or posted a sign about it, instructing interested participants to contact the Principal Investigator. Our parameters included only that participants be “flood affected,” and we left that up to interpretation. In the end, 39 participants had residences that flooded in 2013. Interviews averaged 90 minutes and took place at a public space in the participant’s neighborhood, or in a dedicated space at the university. To thank participants, we offered a $50 gift card to RONA, a Canadian home improvement store. A third-party locally based transcriptionist then transcribed recordings verbatim.

We asked participants various questions about their views on development in risky or flood-prone areas. For this section, participants received the prompt:

The next set of questions asks you about disaster risk—how decisions made by government, the insurance industry, and individuals—can place people at greater risk. In answering these questions, we want you to think about the best ways to protect people and their property from events like the 2013 flood.

Other sections also elicited comments about these issues. The Human Research Ethics Board at Mount Royal University approved the study, and all participant names are changed to pseudonyms to ensure confidentiality.

Although many interviewees discussed their demographics, we did not ask demographic questions specifically. This is because we conducted a survey of the 26 flood-affected neighborhoods only one year earlier (see Gray-Scholz et al. 2019; Haney 2018; Haney and Gray-Scholz 2020; Milnes and Haney 2017). That survey indicated that the neighborhoods were affluent (median income $100,000- $109,999), white (over 90%), educated (63% holding a Bachelor’s degree or higher), and homeowners (over 75%). Thus, the following analyses can be understood to reflect the perspectives of a wealthy class of individuals, living in a wealthy city. Within the sample of 40 interviewees, 20 identified as men, and 20 identified as women. Thirty-five of the 40 participants provided their age, resulting in a mean age of 52, and a median of 55.5, which is slightly higher than, but in the same ballpark as, systematic surveys from Calgary’s flood affected neighborhoods (i.e., see Haney 2019 who found a mean of 48).

Data were analyzed in NVivo 11 using qualitative techniques referred to as “descriptive” and “pattern” coding (Maxwell 2005; Miles and Huberman 1994). First, the author open-coded participants’ responses to identify analytic patterns and themes (Warren and Karner 2010:218–19). These themes were put into different categories (Phillips 2014). Second, these categories were analyzed to identify patterned relationships across categories to determine similarities and differences in the themes. Responses were coded by one research assistant, and then by the author, to ensure inter-coder reliability. In grounded theory fashion, arguments were built by creating an ongoing exchange between the data categories and existing theory in environmental sociology, urban sociology, and the sociology of disaster, in an effort to build upon and reconstruct this existing theory (Glaser and Strauss 2017)—an approach allowing for both parsimony and scope of analysis (Phillips 2014:110).

## Findings

I remember thinking about the community of Chaparral and down in that area where people are building right down in the valley and I thought, ooh, that looks like a really dumb place to buy a house even though I am sure they are gorgeous houses and everything. (Mary-Jean)

The analysis that follows will accomplish three central tasks. First, it explores who Calgarians believe is responsible for creating—and ultimately for curbing—risks. Second, it analyzes how participants discussed two alternative strategies for mitigating risk: on the one hand favoring structural mitigation (levees, dams, or property-level mitigation), while on the other, advocating for mandatory disclosure laws. Third, it analyzes participant beliefs that we have committed to development in flood-prone areas, and thus, we are too late to curb risk.

### Who Should Be Responsible for Mitigating Risk?

#### Developers: The invisible hand of the market

Many flood-affected residents talked extensively about the role of developers in continuing to create risk through irresponsible practices, in addition to developers’ lack of concern over the well-being of residents who will, as a result, occupy a precarious location. Mary-Jean says, “I think developers have a responsibility because they are the ones who are going to profit when those lots are sold, so they should take the responsibility to make it less likely to flood.” For several participants, the experience of the flood made them adopt this more critical stance toward development. Wayne adds:

So after the flood I am always thinking, “What are these developers thinking of?” You are going to build four skyscrapers and you are right on the edge of the bridge, right? So those are going to fill up if anything ever happens . . . Calgary has got to fix the problem and make sure it never floods again.

Several participants bemoaned the lack of constraints for developers to pursue projects in flood-prone areas of the city. Irene believes “developers get away with a lot of shit that they shouldn’t be getting away with.” Others suggested that developers’ lack of concern for resident safety occurs because they are removed quickly from financial liability for flooding (i.e., one year). Matthew echoes this sentiment; “We had a lot of developments in Calgary where the developer’s only responsible for I don’t know a few months or a few years after the development. . . . I can’t remember what it is but it’s not long enough.” Matthew adds that he believes developers have full information about flood risk but conceal this information from would-be homebuyers; “Cause the consumer won’t know . . . And the developer you know in some cases knows. Yeah. Private developers. Absolutely. They should have responsibility.”

Several Calgarians faulted developers for *how* they develop when they build near rivers. According to Roxanne, who immigrated to Canada from Britain:

I mean thinking about it in the UK we don’t have a basement at all . . . So coming here and having basements that you actually live in are like, “Oooh,” and then when I thought about it afterwards, it is like you have these place underground right next to a river? That is insane!

Many approached the role of developers with a sense of futility. For them, developers will pursue profit above all and will oppose regulations. As a result, these residents clamored for the government to regulate developers. Nicole asserts, “I think it is the city who is responsible to map it and say, ‘Okay, you are a developer, and there is this land but you are not allowed to build a residential area.’”

More bluntly, Rachel adds:

The developers can only do what they’re allowed to do. They can’t build where the city says they can’t. They can’t build where the province says they can’t. So it’s not the developers—they don’t give a shit . . . It has to be government who says it can’t be done.

Several other participants made statements that developers “don’t care” about the risk for homeowners. For some, this also meant that developers are blameless in the creation of risk. According to Scott, “You can’t blame the developers, they are just . . . in there to make bucks, right? And if the city says you can build there then bingo . . . They make a pile and they will.” In short, many participants favored stronger land use regulations because they believed that developers were both unable and unwilling to voluntarily limit development activities in risky areas.

#### Government: The courage to stand up to developers?

Because residents did not see private developers as willing nor capable of making land-use decisions that will mitigate risk, many felt that it falls upon government to regulate private developers, and to mandate where new residential development can and cannot take place. To do that, they feel that municipal government—currently viewed as weak and ineffectual—needs courage to stand up to developers. As Jackie states, “I think they have to have legislation in place to say at some point in time, ‘No, developer, you can’t put in a neighborhood there unless you do A, B and C.’” Scott, in saying that he would never again choose to live by a river, added:

I think it is a mistake. The river needs room and . . . the city should not allow it first off. It is the government’s fault. I believe . . . city government allowed that development to occur and the developers did it, so I am very, very unhappy about that.

Likewise, Gary believes that government actors need to “have the balls to say no, and most of them won’t.” When prompted about why they lack “the balls” to regulate developers, he adds “It is all about money.” According to Gary, the money developers wield over politicians influences municipal decisions. In agreement, Scott says, “To hell with the developer and that is what the government has to do. To hell with the developer.”

Developers’ perceived influence over municipal leaders leads some, like Leila, to question the efficacy of representative democracy. Leila agrees with Gary and Scott, adding that by ignoring the will of the people, government is “negligent in their responsibility to the citizens.” By lobbying government to allow building in high-flood-risk areas, Irene believes that “developers get away with a lot of shit that they shouldn’t be getting away with.” Many participants felt that these conversations were taking place between developers and governments, not in spaces where citizens might be consulted.

For participants, government holds the best information about flood risk, and therefore has a responsibility to act. Nancy voices this perspective by saying that city government should shoulder greater ethical burden because they have the fullest information; “at least they’re aware . . . I mean if the information is there, I don’t think they should be developing there.” Frank shares this view, adding that if development is allowed, then developers should carry the corresponding financial liability when housing developments flood:

they should control that and say, “if you are going to allow a developer to develop a whole neighborhood on a flood plain or a swamp . . . then yeah, they should pay for it,” I think, because it never should have been allowed.

Matthew points out that the long-term costs of building in floodplains make such approvals costly for local governments. Advocating for more restrictions, he says developers “can’t develop places where it’s just going to be torn down in 50 years or even 100 years. Think about it. And government, they should help. They should tell private developers maybe. (laughing).” As laughter can be used to indicate discomfort (Nairn 2005), Matthew’s laughter might suggest that he thinks his own proposed solution is unrealistic.

Although nearly all the flood-affected residents clamored for more government regulations on development, there was not complete unanimity. Bryan notes the economic fallout such limitations might provoke, particularly for homeowners rebuilding, renovating, or expanding homes on their existing flooded properties. When asked about granting building permits in flood-prone areas, he said:

they absolutely have to give those permits . . . because they will bankrupt people, and the city of Calgary would leave itself exposed to such huge legal action that they would be bankrupt. So they can’t not give permits. They can finesse that so that you’re building better and more suitable and more durable, but they can’t not give permits, it would be financial suicide for the city.

#### Taking personal responsibility

Given the support for individual responsibility, and corresponding desire for fewer regulations and smaller government in Alberta, it is unsurprising that some participants placed the onus for flood risk mitigation on individuals—not on developers nor government. According to these perspectives, prospective homebuyers should do their research and should avoid living in flood-prone places. Caleb says, “Well I think people can live wherever they want, but I think they have to carry that risk.” Bryan adds that even discussing responsibility for protecting people from flood risk “is really fraught with problems, because it harkens back to private property,” which he feels is sacrosanct. He then says that homeowners make their own choices and protecting them from risk is “really nobody’s responsibility” but their own.

Several participants argued that, living by the river, the possibility of flooding should be “common sense.” According to Caleb, “people have to take some responsibility for themselves, you can’t just go in totally blind and say, ‘I know there is a river here but I am going to build anyways.’ You got to use some common sense.” Rachel refers to this common sense as “instinctual”; “Like if you’re anywhere near the river it should just be instinctual . . . You know? I know the risk.” William feels similar: “Of course, I guess being that close to a river it has to be in the back of your mind that it could happen.” Sociologists, however, are critical of the existence of common sense, arguing that such taken-for-granted knowledge is culturally specific, contextually dependent, and reliant on processes of socialization (Watts 2014).

### Not into Limiting Development? Two Divergent Solutions

#### Engineering away the risk

Rather than restrict development, the usual approach preferred by all levels of government to curtail flood risk has been structural mitigation efforts. Although research has revealed the futility of attempting to engineer away flood risk (Brody Zahran, Highfield, and Grover 2007; Freudenburg et al. 2008), several participants voiced confidence in these efforts. Nicole says, “I think it would be a very good idea if the money would be invested better on some kind of flood-proof wall.” Likewise, Emily says she feels mostly comfortable with new homes going in flood-prone areas, but developers should:

Make it higher. Make it safer. Whatever—put up the concrete walls or whatever you need to do to make it safe. If you really wanted to sell that for development—make it safe—or make the developers make it safe.

Comments by both Emily and Nicole assume that homes in flood-prone areas can be made entirely safe if protected by a strong enough or tall enough wall.

Some homeowners argued that government should be more proactive about building structural protections. Mary-Jean says, “The province should be responsible for doing the dams, or the enforcing or whatever is needed in order to prevent it from happening as well.” According to Emily, “Either you bring in a lot of dirt and don’t build basements and make it safe . . . The city should make sure that people live in safe places!”

And some residents argue that the city should not necessarily regulate or limit *where* we develop housing but should instead focus on *how* we build housing. According to Jocelyn:

I think it makes more sense to put in really well-designed homes and go bigger than you even thought possible. Make it so you can’t even have a ground floor, or like just parking under your house . . . where the house is up on stilts and the car goes underneath.

Dave adds:

The government should have a lot to say about that, that’s why we get building permits that’s why we get development permits so there should be, I think, so if you’re gonna build a house here you gotta build it on stilts, it might not look like nice but I mean that the kind of thing we almost have to do.

Endemic to these approaches is the idea that structural mitigation is key to conquering flood risk. Some argue for larger community-level projects—dams or levees—while others argue for property-level flood mitigation projects—like not allowing basements or building houses on stilts—but these approaches share their belief that flood risk can be engineered away and, in doing so, imply that development will or should continue. Few participants discussed any specific government failures to mitigate flood risk, and many projects were still underway (two years post-flood). Many discussed the Province of Alberta’s proposed Springbank dam and off-stream reservoir, which was slated to be built west of the city on rural land that would need to be bought out. They voiced hope that this project might make their properties safer, but as of summer 2021 (six years since the interviews), it has not been constructed. If completed, it might provide some mental—if not hydrological—security to residents and aid in the process of placemaking in these newer communities (see Coaffee 2013; Wu and Hou 2020).

#### The need to inform?

One question asked residents specifically if potential homebuyers should be made aware of their home’s location in a flood-prone area. Although there was some dissention, homeowners largely believed that real estate agents should shoulder this responsibility. Dave discussed his brother, who bought a home and did not think about flood risk:

why would somebody not warn him when he went to buy there? And that’s quite often what happens, you see, you end up not knowing that especially if you’re not from around there, so that’s the kind of stuff I think the government should control.

Peter agrees that a homebuyer:

should have total disclosure. You know, if I was to sell my house, we would tell the people that, yeah, the city flooded in 2013 . . . I think that it’s the responsibility of realtors to make sure that people buy and know what they’re getting into.

Even if that disclosure would chase away potential buyers or lower the sale price, Peter maintains that he would do so out of fairness.

Angela also homed in on the fact that many homeowners may not understand their actual levels of flood risk from a simple disclosure, so she added that realtors should “not just say that but explain what it means.” Tasha echoed the need for more context. She knew she lived in a floodplain, but not the real, actualized flood risk:

I only knew I was in a flood plain, and then after the flood I was told I was in a flood fringe and I never, ever heard the word in my life . . . . I have lived here for 42 years and I have never heard of “flood fringe” ever . . . so maybe realtors should be more upfront that, “this is zoned ‘flood plain’ and this is zoned ‘flood fringe.’”

These examples represent a vast majority of residents who felt that homebuyers should be made aware prior to the purchase.

A few residents, however, did not think it was advisable to inform homebuyers, as that responsibility should fall onto the individual as a “buyer beware” scenario. According to Allan, this has to do with the complexity of flood risk and experts’ inability to understand it, let alone communicate it effectively; “No, I think the onus is on [the individual]. The problem with that is what is the flood risk, right? Really what is the flood risk? You can’t quantify that realistically, right? It is a roll of the dice and you are gambling.” Similarly, Bryan says:

So, disclosure laws are pretty clear right now. You can’t sell a flooded property to somebody and not tell them. To tell them about future risk is kind of like: this house might burn down. That tree could fall on you. This could flood again. So, buyer beware, they should know that they’re buying on a flood fringe.

Those who supported a mandatory informing law are also noteworthy because such a policy would almost surely lower their own property values, as homeowners who already occupy these spaces. Nonetheless, many spoke convincingly about how they wished they had been informed before they bought.

### Is It Too Little, Too Late?

Many participants expressed marked confusion about responsibility, and which parties could be held responsible for which decisions, while several others felt fatalistically that it is now too late to curb development.

In grappling with the complexity of floodplain management, Irene contends that if the city allows housing to be built in flood-prone areas, then insurance companies should “damn well cover it.” This comment implies that municipalities should have some control over private sector insurers, influence they do not normally wield. Christian expressed confusion when asked who should be responsible for ensuring that Calgarians do not live in flood-prone areas, saying:

I don’t think they ummm they worked, they worked on it consciously. I may be wrong. But I think there are so many other things to work on. Uhhh, the other realities, you know say developers against the environmental lobby. That’s not fair either.

Such hard-to-interpret responses reveal participants’ struggles to understand the complexities involved in non-structural forms of flood-risk mitigation. William argues that flood insurance ought to be mandatory in flood-prone areas of the city: “it would be nice to see that as part of the mandate for allowing permits for developers to build in those areas that are flood-prone.” This ignores the fact that residential flood insurance was not available in Canada until 2015, is still widely unavailable (Thistlethwaite 2017), and that homeowners carry unrealistic expectations for how low-cost this coverage—which is not government-subsidized, unlike in the United States—should be (Thistlethwaite et al. 2017). While laws about holding mandatory insurance may sound attractive, participants may not have understood the institutional and financial barriers to implementing such policy.

Several participants felt the questions about flood risk creation were too-little-too-late as Calgary has fully committed to building in flood-prone areas. The homes are built and neighborhoods established, so there is now nothing that can be done. Tasha notes, “there is not a lot of land left in Calgary [where we might] say that is a vulnerable area and you should not live in it. So the dirty deed has been done.” Allan felt that so much of the city’s population lives in flood-prone areas that talk of keeping people away from risk is now inane or futile; “well unfortunately it is a little late now, you know?” This view ignores new and ongoing development in flood-prone areas, like Riverstone (see Introduction). It also rejects possible solutions such as “managed retreat” (Koslov 2016), which can be used to move populations from vulnerable places. Rachel points out that this approach can be costly. When asked about areas with higher flood risk, she responded by saying that removing existing development would be impossible; “you’re gonna have to buy out 700, 800, 900 homes. There’s just a shit-ton of homes that are in flood fringe.” Peter also saw this approach as undesirable, saying “Half the city’s built on a riverbank . . . . So you’re not going to force all those people to pack up and move.” And lastly, when asked how to keep people from living in vulnerable places, Edward added:

Well they already do, so . . . and it is not just people, I mean it is . . . Calgary, downtown, headquarters of major national and international companies [are] in a flood plain of the river. I can’t imagine what cost it would be to the city, to the local economy, to the provincial economy, to the national economy if nobody was allowed to live in a flood plain, if no major businesses were allowed to operate in the flood plain. . . . I don’t think that you could do it, frankly.

Discussing flood risk in their community forced many participants to confront complex problems of global, systemic risk. William acknowledges the need for multiple stakeholders to maintain a common operating picture and collaborate to decrease risk, while at the same time acknowledging the difficulty:

I suppose that all depends on the insurance that is available as well. If there . . . I mean it is kind of hard to manage if you are issuing permits, to ensure that people have the proper insurance to cover a flood, but I mean, developers are only on the hook for so long after developing an area. I guess in an ideal world no, they shouldn’t allow permits for flood prone areas, but then just due to normal population growth it is kind of hard to avoid it.

Similarly, Christian has begun to think about just how many people live in vulnerable places. He feels that prohibiting development in vulnerable places “would include ‘tornado alley’ near Edmonton where you have more tornados than there are in southern Alberta. It could also mean possibly other areas. I can’t think of what I mean by that but you know, geographical problems.” Gary mused that it is impossible for us all to live in the middle of Saskatchewan to avoid risk. He paused and added:

Regina? Built up on a river. Saskatoon? Built on a river. Edmonton? Built on a river. Vancouver? Built on the ocean. Tuktoyaktuk? Built on the ocean . . . Those are the natural trade routes that go back hundreds of years and that is why they are there.

Allan similarly concludes, “It is not just Calgary, it is everywhere that people live in disaster areas . . . You can’t change that.”

## Conclusion

Risk is proliferating in North American cities, and new neighborhoods are being planned and constructed in close proximity to rivers. As the “growth machine” (Logan and Molotch 1987; Molotch 1976) pushes for unabated development, it is important to examine how vulnerable residents understand these decisions and to renew the conversation about how we might keep people and property away from hazards.

Using the case of Calgary, Alberta, this article investigated several potential avenues, including the possibility of placing limitations on new housing in flood-prone areas. By interviewing residents—nearly all of them homeowners—who had recently been affected by flooding, this analysis demonstrated how they conceived of risk and allocated responsibility for protecting residents. In doing so, it highlights ongoing debates about the politics and motivations of property owners, both during normal times (Becher 2015; Dupuis and Thorns 1998; Ruef and Kwon 2016) and in situations of risk and disaster (Thistlethwaite et al. 2018; Zavar 2015). At the same time, it highlights notions of trust and the expectations that residents hold of municipal government in protecting property value (Becher 2015; McCabe 2016; Poortinga and Pidgeon 2003). Furthermore, it helps us better grasp how members of conservative communities, who normally reject regulation and vilify “activist governments,” nevertheless use government and select land-use regulations to protect their property (see Hochschild 2016). Doing this work in relatively privileged communities reveals the barriers at play in cities struggling to adapt to a changing climate (Goodell 2017; Smiley 2017; Stone 2012), as wealthy residents often serve as obstacles to climate action (Page, Bartels, and Seawright 2013), while living some of the most highly consumptive lifestyles (Harlan, Pellow, and Roberts 2015; Solarin 2019).

Participants varied in their attribution of risk and in their assignment of responsibility, but many agreed that building should be curtailed in areas prone to flooding. A few residents did object to such regulations, owing to “buyer beware” attitudes or the sanctity of private property rights. Those residents often preferred to enact mandatory disclosure laws where homebuyers are informed about a home’s location in a flood zone or, in some cases, preferred only enhanced structural mitigation techniques to protect both existing and new neighborhoods. Virtually none felt that developers should themselves be responsible for curtailing risky development, as they were cast as myopic profit-driven capitalists, who will not voluntarily take actions to protect public safety. Participants typically were not angry about developers’ complacence but resigned to it. Many felt that homes had already been built, and it was now too late to halt development.

The above qualitative analyses reveal, however, that participants tended to discuss only one approach to mitigating risk and fell into three relatively mutually exclusive camps: several argued for more government regulation of building in the floodplain, often along with mandatory disclosure laws informing homebuyers of risk; a second group focused on the need for enhanced structural mitigation efforts, including new dams, levees, and floodwalls; and a third group spoke about the importance of personal responsibility (“buyer beware”), often also expressing feelings that we are too late to curb the creation of new risks. Participants tended to stick to one of these preferred approaches, rather than discussing them in tandem. For instance, several participants feel it is “too little, too late” to mitigate flood risk, and then also discussed the need for homeowners to take personal responsibility by not buying homes in flood-prone areas (i.e., Allen, Bryan, and Rachel), which seems logically quite consistent. One exception to these mutually exclusive approaches is Gary who spoke about the need for government to stand up to developers, but also noted how he felt we were too late to curb risk, as houses had already been built. By and large, however, participants tended to prefer government restrictions on development and/or the need to inform homebuyers, the need for enhanced structural mitigation, or the need for homeowners to own the risk, in fairly mutually exclusive camps. These findings suggest a certain disagreement, whereby residents of flood-prone communities lack common ground and preferred approaches to flood risk mitigation, and differ in their understandings of continued floodplain development.

The lack of consensus on who should shoulder the responsibility to curb growing flood risk likely means that river-adjacent residents will have little collective voice and there may be little movement in any one direction, other than to stay the course. Given that I collected data from flood-affected residents—those perhaps *most* likely to favor restrictions on new development—we may surmise that residents will not likely be able to collectively resist ongoing and future development. Indeed, research shows that even when residents are organized and share a relatively common vision on risk mitigation, advocating to governmental agencies and actors for these desired outcomes can still be challenging (Koslov 2016, 2019). These findings help us better understand how in “hegemonic growth regimes” most citizen groups end up being either pro-growth or neutral, and do not present challenges to the ubiquity of growth (Logan and Crowder 2002). It is also noteworthy that since Calgary’s river communities contain some of the most wealthy residents, many participants either hold informal membership in the “growth elite” (Logan and Molotch 1987; Molotch 1976) or stand to indirectly benefit from continued growth. These vested interests may help us understand why residents stopped short of voicing a uniform call to end floodplain development.

Like all research, the present study carries limitations. The choice to interview residents from these neighborhoods makes sense for post-disaster research, as they had just experienced a catastrophic flood. At the same time, that methodological choice also carries distinct limitations as findings from wealthy hazard-prone residents may not generalize well to residents living near hazards in other cities (who are more likely to be lower-income and therefore may have different concerns and attributions of responsibility); they may, for instance, clamor even more loudly for cessation of development near hazards. They may also be more aware, and more critical, of the city’s “growth elite” as a key driver of risk and vulnerability. In addition, the qualitative data presented above give us little insight into *who* subscribes to these differing preferred approaches to flood risk mitigation. Analysis of gender differences, age, occupation, neighborhood of residence, and even education, provided little that might help us understand how social and demographic factors drive these divergent attitudes and ideas. Future research should look into how many of the above factors (not to mention race/ethnicity, nativity, and other axes of inequality) contribute to and explain different approaches to floodplain development, risk creation, and hazard mitigation.

These findings are particularly noteworthy in Alberta, where many residents subscribe to an individualistic outlook and decry new government regulations as job-killing red-tape. In fact, in 2019 the Government of Alberta appointed a “Red Tape Commission” and a Minister of Red Tape, charged with cutting regulations wherever possible (Maimann 2019). Yet, many homeowners in areas of the city that flooded in 2013 are calling on government to better regulate development and to ensure that developers and/or realtors better educate homebuyers about flood risk. Given that mandatory disclosure rules would presumably harm their property’s value, we might feel sanguine that even residents with much material stake in the status quo nevertheless recommended adopting better policies for flood risk disclosure. However, the real estate industry derides these approaches, labeling them “idiotic” and saying that mandatory informer requirements would “kill the market” (Goodell 2017:97). Such objections suggest a larger problem: “nobody wants to spend money to build a more resilient city because nobody owns the risk” (p. 103). The practices of risk trading, epitomized by the global reinsurance industry (Jarazabkowski, Bednarek, and Spee 2017), as well as industry efforts to influence policy and avoid financial liability, effectively shifts risks onto property owners. At the same time, as the findings demonstrate, a number of participants attributed responsibility for risk to homeowners who, they feel, should know better than to purchase homes in flood-prone areas, effectively devolving the responsibility away from developers and government, and urging more personal responsibility—a finding that should not be surprising in the Alberta context, but does not lead us logically to many promising policy interventions.

The 2015 introduction of private flood insurance has protected a small number of very wealthy homeowners, but will also likely continue to drive development in risky areas (Thistlethwaite 2017), and uptake of these products has been low as most Canadians hold unrealistically low reservation prices for premiums (Thistlethwaite et al. 2017). Although insurance may not be the answer, other ideas such as managed retreat (Koslov 2016) hold potential, particularly because they are resident driven approaches to adapt to a changing climate. Thus, managing risk appropriately requires a shift in thinking. For instance, in 1994 the town of Chelsea, Iowa voted to move the entire town out of the flood-zone. According to the town’s Mayor, “It used to be that we manage the river and let people go where they want to . . . Now we are trying to manage the people and letting the river go where it wants to” (Steinberg 2000:119). As climate change intensifies, more cities may find themselves making room for the river. For now, asking municipal governments to stand firm against monied development interests, and to restrict new housing in flood-prone areas, is a pragmatic approach. After all, the first step in getting out of a hole is to stop digging.
